# Cardiovascular and Metabolic Adverse Events of Endocrine Therapies in Women with Breast Cancer: A Disproportionality Analysis of Reports in the FDA Adverse Event Reporting System

**DOI:** 10.1002/cam4.70548

**Published:** 2024-12-30

**Authors:** Shaimaa Elshafie, Lorenzo Villa‐Zapata, Randall L. Tackett, Iman Y. Zaghloul, Henry N. Young

**Affiliations:** ^1^ Department of Clinical and Administrative Pharmacy, College of Pharmacy University of Georgia Athens Georgia USA; ^2^ Central Administration for Drug Control Egyptian Drug Authority Cairo Egypt; ^3^ School of Pharmacy MCPHS University Boston Massachusetts USA

**Keywords:** adverse drug reactions, breast cancer, cardiovascular risk, disproportionality analysis, endocrine therapy, FAERS database

## Abstract

**Introduction:**

Emerging evidence suggests potential cardiovascular toxicities from oral endocrine therapies (ETs); however, results are conflicting. This study comprehensively examined adverse reactions of ETs and investigated cardiovascular and metabolic safety signals within the FDA Adverse Event Reporting System (FAERS).

**Methods:**

Reports in the FAERS through December 2023 were analyzed for documented reactions to tamoxifen, letrozole, anastrozole, and exemestane in female breast cancer patients. Standardized queries were used to identify cases of cardiovascular (myocardial infarction, heart failure, arrhythmia, stroke) and metabolic (hypertension, dyslipidemia, hyperglycemia) disorders. Descriptive and disproportionality analyses were performed to assess reports and detect safety signals.

**Results:**

Among 14,327 unique ET‐related reports, arthralgia (*n* = 1873 events) was the most prevalent reaction. We identified 2170 cardiovascular and 2252 metabolic events associated with ETs. Letrozole had the highest reporting rate of cardiac arrhythmia (7.7%) and showed positive signals for both arrhythmia (reporting odds ratio [ROR] = 2.2; 95% confidence interval [CI]: 1.8–2.5) and myocardial infarction (ROR = 1.9; 95% CI: 1.4–2.6). We also observed a significantly increased risk of heart failure with letrozole (ROR = 1.3; 95% CI: 1.1–1.6) and stroke with tamoxifen (ROR = 1.7; 95% CI: 1.5–2.1). Only anastrozole was significantly associated with metabolic dysfunctions with a notable hyperglycemia reporting rate of 12.2%.

**Conclusion:**

Our findings provide valuable evidence on common reactions as well as controversial cardiovascular and metabolic abnormalities associated with the real‐world use of ETs for breast cancer. Ongoing benefit–risk assessment and close monitoring of cardiac function during treatment, particularly in high‐risk women, are warranted to optimize cancer outcomes while minimizing cardiovascular injury.

## 1 Introduction

Adverse drug reactions (ADRs) are concomitant hazards of therapeutic use that may lead to treatment discontinuation, morbidity, mortality, and substantial economic costs [1, 2]. Historical iatrogenic disasters have triggered careful consideration and surveillance of pharmaceutical products' adverse reactions; [3] however, this response has not been as rigorous in cancer treatment. Proof of maximum survival efficacy often takes precedence over potential toxicities of anticancer therapies, even though these toxicities can be serious and life‐threatening posing a critical concern to patients [4]. Additionally, insights into the ADRs of oncology medications emerge primarily from clinical trials which are usually limited by the small number of participants, variability of regimens, exclusion of certain patient populations, and short follow‐up durations [5, 6].

The limited information on ADRs along with the increasing life expectancy of cancer patients has prompted national health authorities to expand their focus to integrate safety data from real‐world clinical practices [7]. This approach aims to address latent and long‐term issues of anticancer medications, optimize treatment outcomes, and improve patients' quality of life during their illness and forward into survivorship.

Currently, the number of overall cancer survivors in the United States (US) exceeds 18 million with female breast cancer ranking at the top accounting for over 4 million survivors [8]. Oral endocrine therapies (ETs) have been a fundamental component of breast cancer treatment in both the curative and palliative care settings [9]. Tamoxifen (TAM) marked the first ET approved by the US Food and Drug Administration (FDA) in the late 1970s and was followed later by aromatase inhibitors (AIs), including anastrozole (ANA), letrozole (LET), and exemestane (EXE) [10].

Many adverse reactions to ETs are still underreported, rarely examined, or completely unrevealed. Recent controversial and inconclusive evidence has linked some ET regimens to serious cardiovascular complications [11, 12]. These potential risks are particularly concerning given the observed high rate of heart‐related deaths among breast cancer survivors [13]. The prevalence of cardiovascular problems in these women is explained by the interplay of risk factors (e.g., age and obesity) between cancer and cardiovascular diseases, and the cardiotoxic effects of typical breast cancer treatments (e.g., radiotherapy and anthracycline chemotherapeutics) [14]. In addition to patient vulnerability, recent expert recommendations to extend ET courses up to 10 years for more favorable clinical outcomes may subsequently increase the occurrence of adverse events [15].

The long‐term administration of systemic ETs emphasizes the importance of a detailed assessment of overall adverse reactions and the evaluation of emerging cardiovascular issues. Utilizing data from pharmacovigilance platforms can complement the current evidence and bridge the knowledge gap in the cardiovascular safety profile of ETs. The purpose of this study was to identify the commonly reported adverse events of each ET and investigate safety signals of specific cardiovascular events among female breast cancer cases within the FDA database.

## 2 Methods

This study adheres to the latest Reporting of A Disproportionality Analysis for Drug Safety Signal Detection Using Individual Case Safety Reports in PharmacoVigilance (READUS‐PV) guidelines to ensure transparency and comprehensiveness in our reporting [16].

### 2.1 Data Source

This retrospective pharmacovigilance study was designed to evaluate adverse reactions attributable to the real‐world use of oral ETs (TAM, LET, ANA, and EXE) within the FDA Adverse Event Reporting System (FAERS). The FAERS is a publicly available database that supports the FDA's surveillance of the safety of pharmaceutical products in the post‐marketing phase. This tool provides information on reports submitted to the FDA by manufacturers, healthcare professionals, and consumers.

### 2.2 Data Collection

The FAERS database was searched using both brand and generic names of each medication under study. All retrieved reports of ET reactions that occurred exclusively in females through the end of 2023 were downloaded from the FAERS dashboard [17]. Data files were imported into Microsoft Excel spreadsheets for initial data management. The reason for medication use was limited to malignant breast tumors (of any subtype or stage), and cases with other clinical indications were excluded. Reports that involved irrelevant reactions (such as administration errors, quality complaints, treatment ineffectiveness) or contained a non‐ET regimen as a co‐suspected product were also removed to eliminate the confounding effect of concomitant medications. Duplicate entries were identified through identical patient age and weight, reported events, as well as the date and country of event occurrence, and were subsequently removed. The final list of cases was cross‐checked and verified by a second researcher to ensure data integrity and reliability.

### 2.3 Outcome Definitions

We reported the most frequent ADRs (5% or above) for each therapy based on the Preferred Terms (PTs) in the Medical Dictionary for Regulatory Activities (MedDRA version 27.0). The PTs are used to define terminologies for reactions in the database. Cardiovascular‐related adverse events were extracted and grouped according to the Standardized MedDRA Queries (SMQs) which are validated composites of multiple terms related to a specific medical condition. Primary complications of interest included myocardial infarction, heart failure, arrhythmia, and stroke (cerebrovascular disorders). We also investigated metabolic dysfunctions that increase the risk of adverse cardiovascular outcomes, including hypertension, dyslipidemia, and hyperglycemia (weight changes and blood glucose abnormalities). A detailed definition of the SMQs used in our study is available in the Supplementary Appendix—Data S1.

### 2.4 Statistical Analysis

Descriptive statistics (counts and percentages) were calculated for cases and reported reactions by treatment regimen over the study period. Reports were characterized by the mean age of patients, reporter type, seriousness, event outcomes, and the region where the event occurred. The frequency for each category of common reactions was estimated and tabulated. Disproportionality metrics (reporting odds ratio [ROR], proportional reporting ratio [PRR], and information component [IC]) and their corresponding 95% confidence intervals (CIs) were computed for each regimen alone in comparison to all other ETs (reference group) across the abovementioned outcomes using R software version 4.3.2. A signal means a possible causal relationship between a medication and an adverse event that suggests the need for further action to prevent harm [18]. Safety signals were deemed positive or significant when the following criteria were met altogether: (a) the number of co‐occurrences was ≥ 3 and the lower limit of the 95% CI of the ROR exceeded 1, (b) the PRR was ≥ 2 with an associated chi‐square ($X^{2}$) of ≥ 4, and (c) the lower limit of the 95% CI of the IC (${\mathrm{IC}}_{025}$) exceeded 0 [19, 20, 21].

Applying simultaneous concordance of these three algorithms which conform to different methodologies (frequentist and Bayesian statistics) allowed for the reduction of false‐positive associations and led to accurate and reliable signal detection. These conservative considerations originated from the variability in disproportionality findings based on the type of measure used and the lack of a single well‐accepted standard index for signal identification [22]. Similar approaches were used in other previous studies [23, 24].

## 3 Results

### 3.1 Reports Characteristics

**FIGURE 1:**
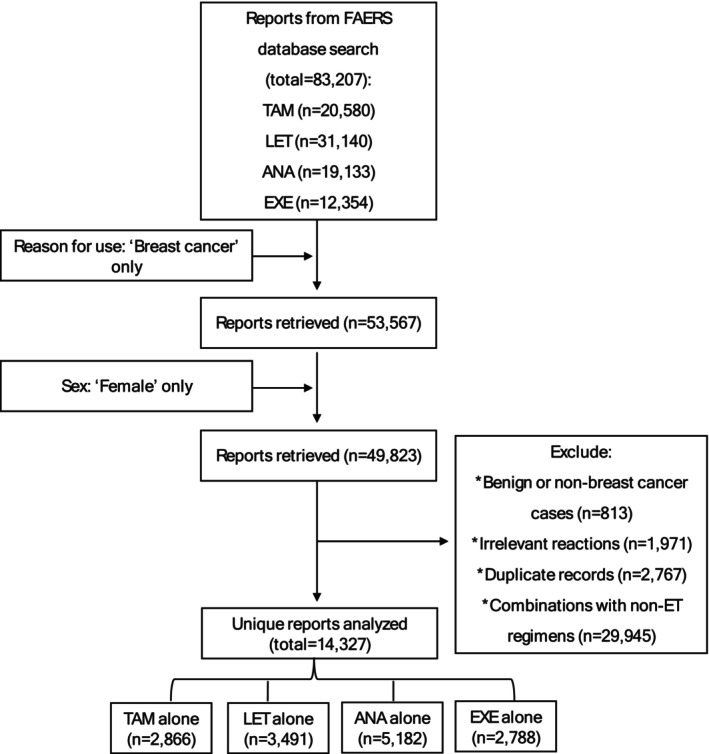
Study flowchart. ANA, anastrozole; EXE, exemestane; FAERS, FDA Adverse Event Reporting System; LET, letrozole; TAM, tamoxifen.

Out of more than 83,000 reports in the FDA database, we identified and analyzed 14,327 unique safety reports pertaining to ETs in women with breast cancer (Figure 1). The ANA regimen had the highest number of reports which accounted for more than one‐third of the total (*n* = 5182; 36%) and was followed by LET (*n* = 3491; 24%). Approximately half (49%) of all reports were documented by healthcare professionals, whereas 39% were documented by consumers. Contributions to the submissions from the US were comparable to those from other countries.

Patients who received TAM were the youngest (61 years on average; standard deviation = 13) while the mean ages of those on AI therapies were slightly older (from 65 to 67 years; standard deviation = 11). Few cases were defined as metastatic or advanced stage. Most reactions were identified as serious by the submitters. Hospitalization represented 36%, 29%, 25%, and 18% of ADR outcomes of TAM, LET, EXE, and ANA respectively. Table 1 summarizes the main characteristics of the analyzed reports.

**TABLE 1:** Characteristics of reports of ET reactions in the FAERS (up to 2023).

Attribute	Regimen			
	TAM	LET	ANA	EXE
	*N* = 2866	*N* = 3491	*N* = 5182	*N* = 2788
Patient				
Age, mean years (SD)	61 (13)	65 (11)	66 (11)	67 (11)
Age unspecified	395 (14)	830 (24)	869 (17)	315 (11)
Reporter				
Healthcare professional	1552 (54)	2287 (65)	1888 (37)	1285 (46)
Consumer	613 (21)	1034 (30)	2551 (49)	1387 (50)
Unspecified	701 (25)	170 (5)	743 (14)	116 (4)
Indication				
Metastatic cancer	47 (2)	343 (10)	159 (3)	328 (12)
Reaction severity				
Serious	2649 (92)	3153 (90)	3160 (61)	1974 (71)
Nonserious	217 (8)	338 (10)	2022 (39)	814 (29)
Reaction outcomes^a^				
Death	168 (6)	278 (8)	125 (2)	130 (5)
Life‐threatening	207 (7)	138 (4)	126 (2)	69 (3)
Hospitalization	1040 (36)	995 (29)	928 (18)	687 (25)
Disability	305 (11)	217 (6)	363 (7)	112 (4)
Required intervention	549 (19)	19 (0.5)	270 (5)	12 (0.4)
Congenital anomaly	8	0	2	1
Other outcomes	1145 (40)	2181 (62)	1939 (37)	1363 (49)
Country of event occurrence				
US	403 (14)	749 (21)	2946 (57)	1406 (50)
Other	979 (34)	2358 (68)	1454 (28)	943 (34)
Unspecified	1484 (52)	384 (11)	782 (15)	439 (16)

*Note:* All values are expressed as *n* (%) unless otherwise noted.

Abbreviations: ANA, anastrozole; ET, endocrine therapy; EXE, exemestane; FAERS, FDA Adverse Event Reporting System; LET, letrozole; SD, standard deviation; TAM, tamoxifen; US, United States.

^a^ A report could indicate more than one outcome.

### 3.2 Common Events

Data that were collected from the FAERS contained 7417 safety issues related to ETs. Among all events, the number of commonly reported reactions ranged from as low as 7 for TAM to up to 31 for ANA with an overall 35 unique common adverse outcomes for all therapies combined. Higher‐grade complications included depression, dyspnea, pulmonary embolism, and uterine polyps. Depression was specifically reported for ANA at a rate of 11%, while pulmonary embolism and uterine polyps were exclusive to TAM with a frequency of 6% each. Dyspnea occurred with all therapies and ranged from 5% to 7%.

Arthralgia or joint discomfort was the most prevalent symptom (*n* = 1873; 25%) across all ETs; however, the rate differed by regimen: 7% for TAM and 19%, 22%, and 50% for LET, EXE, and ANA respectively. Other consistently reported adverse reactions across the four therapies included hot flushes, fatigue, and dyspnea. The highest frequencies were generally observed in ANA users. Table 2 displays the common events for each therapy.

**TABLE 2:** Commonly reported reactions for each ET regimen.

Events	Anastrozole		Exemestane		Letrozole		Tamoxifen	
	*N*	%	*N*	%	*N*	%	*N*	%
Total	2025	100	1483	100	2208	100	1701	100
Arthralgia	1005	49.6	329	22.2	421	19.1	118	6.9
Hot flush	564	27.9	137	9.2	158	7.2	87	5.1
Fatigue	385	19.0	175	11.8	218	9.9	90	5.3
Pain in extremity	327	16.1	105	7.1	143	6.5	—	—
Weight increased	305	15.1	75	5.1	—	—	93	5.5
Alopecia	293	14.5	106	7.1	—	—	—	—
Insomnia	285	14.1	117	7.9	—	—	—	—
Nausea	280	13.8	105	7.1	152	6.9	—	—
Headache	278	13.7	109	7.3	129	5.8	—	—
Bone Pain	268	13.2	94	6.3	151	6.8	—	—
Pain	242	12.0	109	7.3	167	7.6	—	—
Asthenia	239	11.8	95	6.4	118	5.3	—	—
Myalgia	230	11.4	101	6.8	149	6.7	—	—
Dizziness	181	8.9	101	6.8	127	5.8	—	—
Dyspnea	140	6.9	79	5.3	155	7.0	86	5.1
Fall	128	6.3	—	—	116	5.3	—	—
Diarrhea	124	6.1	79	5.3	—	—	—	—
Depression	222	11.0	—	—	—	—	—	—
Arthritis	215	10.6	—	—	—	—	—	—
Back Pain	194	9.6	—	—	—	—	—	—
Gait disturbance	162	8.0	—	—	—	—	—	—
Oedema peripheral	155	7.7	—	—	—	—	—	—
Hypoesthesia	148	7.3	—	—	—	—	—	—
Paresthesia	144	7.1	—	—	—	—	—	—
Osteoporosis	141	7.0	—	—	—	—	—	—
Hypertension	125	6.2	—	—	—	—	—	—
Carpal tunnel syndrome	120	5.9	—	—	—	—	—	—
Musculoskeletal stiffness	116	5.7	—	—	—	—	—	—
Trigger finger	114	5.6	—	—	—	—	—	—
Cough	111	5.5	—	—	—	—	—	—
Muscle spasms	110	5.4	—	—	—	—	—	—
Malaise	—	—	125	8.4	—	—	—	—
Pruritus	—	—	75	5.1	—	—	—	—
Pulmonary embolism	—	—	—	—	—	—	96	5.6
Uterine polyp	—	—	—	—	—	—	93	5.5

### 3.3 Cardiovascular and Metabolic Events

Among all reports of ETs, we identified a total of 2170 cardiovascular events (15 cases per 100 reports). Heart failure was the most frequent cardiovascular event (*n* = 695; 5%), while myocardial infarction was the least (*n* = 171; 1%). LET had the highest number of adverse cardiovascular effects with cardiac arrhythmia being reported the most at a rate of 8%. In addition, a total of 2252 metabolic events related to ETs were reported. Hyperglycemia was common with the highest rate observed for ANA (12%). Hypertension was rarely mentioned in TAM reports (*n* = 28; 1%). Results of the descriptive analyses of cardiovascular and metabolic reactions are listed in Table 3.

**TABLE 3:** Frequency of cardiovascular and metabolic disorders associated with ETs.

Event^a^	Regimen				Total *N* = 14,327
	TAM *N* = 2866	LET *N* = 3491	ANA *N* = 5182	EXE *N* = 2788	
Myocardial infarction	37 (1.3)	65 (1.9)	38 (0.7)	31 (1.1)	171 (1.2)
Cardiac failure	97 (3.4)	206 (5.9)	252 (4.9)	140 (5.0)	695 (4.9)
Arrhythmia	93 (3.2)	269 (7.7)	212 (4.1)	99 (3.6)	673 (4.7)
Stroke	188 (6.6)	162 (4.6)	145 (2.8)	136 (4.9)	631 (4.4)
Hypertension	28 (1.0)	141 (4.0)	242 (4.7)	101 (3.6)	512 (3.6)
Dyslipidemia	43 (1.5)	67 (1.9)	151 (2.9)	42 (1.5)	303 (2.1)
Hyperglycemia	220 (7.7)	307 (8.8)	632 (12.2)	278 (10.0)	1437 (10.0)

*Note:* All values are expressed as number of cases (%).

Abbreviations: ANA, anastrozole; ET, endocrine therapy; EXE, exemestane; LET, letrozole; TAM, tamoxifen.

^a^ Grouped by Standardized MedDRA Queries (SMQs).

The disproportionality analysis showed safety signals for arrhythmia [ROR = 2.2 (95% CI: 1.8–2.5), PRR = 2.1 ($X^{2}$ = 93.3) and ${\mathrm{IC}}_{025}$ = 0.5] and myocardial infarction [ROR = 1.9 (95% CI: 1.4–2.6), PRR = 2.0 ($X^{2}$ = 17.5) and ${\mathrm{IC}}_{025}$ = 0.3] from LET therapy. A significantly higher likelihood of stroke development was observed with TAM (ROR = 1.7; 95% CI: 1.5–2.1) and heart failure with LET (ROR = 1.3; 95% CI: 1.1–1.6). These associations showed statistically significant RORs but did not meet the PRR condition for signal detection. ANA was the only regimen associated with a significantly higher risk of reporting any metabolic disorder, including hypertension, dyslipidemia, and hyperglycemia. Table 4 presents the results of the pharmacovigilance analysis for each ET compared to all other therapies (reference group).

**TABLE 4:** Disproportionality analysis results for cardiovascular and metabolic disorders associated with ETs.

Event^a^	Regimen											
	TAM			LET			ANA			EXE		
	ROR (95% CI)	PRR	${\mathrm{IC}}_{025}$	ROR (95% CI)	PRR	${\mathrm{IC}}_{025}$	ROR (95% CI)	PRR	${\mathrm{IC}}_{025}$	ROR (95% CI)	PRR	${\mathrm{IC}}_{025}$
Myocardial infarction	1.1 (0.8–1.6)	1.1	−0.4	**1.9 (1.4–2.6)**	**2.0**	**0.3**	0.5 (0.3–0.7)	0.5	−1.2	0.9 (0.6–1.4)	0.9	−0.7
Cardiac failure	0.6 (0.5–0.8)	0.6	−0.8	**1.3 (1.1–1.6)**	1.3	**0.1**	1.0 (0.9–1.2)	1.0	−0.2	1.0 (0.9–1.3)	1.0	−0.2
Arrhythmia	0.6 (0.5–0.8)	0.6	−0.8	**2.2 (1.8–2.5)**	**2.1**	**0.5**	0.8 (0.7–0.9)	0.8	−0.4	0.7 (0.6–0.9)	0.7	−0.7
Stroke	**1.7 (1.5–2.1)**	1.7	**0.4**	1.1 (0.9–1.3)	1.1	−0.2	0.5 (0.4–0.6)	0.5	−0.9	1.1 (0.9–1.4)	1.1	−0.1
Hypertension	0.2 (0.2–0.3)	0.2	−2.4	1.2 (1.0–1.4)	1.2	−0.1	**1.6 (1.3–1.9)**	1.6	**0.2**	1.0 (0.8–1.3)	1.0	−0.3
Dyslipidemia	0.7 (0.5–0.9)	0.7	−1.0	0.9 (0.7–1.2)	0.9	−0.5	**1.8 (1.4–2.2)**	1.8	**0.2**	0.7 (0.5–0.9)	0.7	−1.0
Hyperglycemia	0.7 (0.6–0.8)	0.7	−0.6	0.8 (0.7–0.9)	0.8	−0.4	**1.4 (1.3–1.6)**	1.4	**0.2**	1.0 (0.9–1.1)	1.0	−0.2

*Note:* Bold font indicates meeting significance condition.

Abbreviations: ANA, anastrozole; CI, confidence interval; ET, endocrine therapy; EXE, exemestane; ${\mathrm{IC}}_{025}$, lower limit of the 95% confidence interval of the information component; LET, letrozole; PRR, proportional reporting ratio; ROR, reporting odds ratio; TAM, tamoxifen.

^a^ Grouped by Standardized MedDRA Queries (SMQs).

## 4 Discussion

Our evaluation of adverse event reports of oral ETs that have been widely used to treat nonmetastatic and advanced breast cancer (TAM, LET, ANA, and EXE) identified multiple safety concerns. Musculoskeletal disorders specifically joint pain were the most common problems across treatment regimens. We also found evidence of significant toxicities on the heart and blood vessels associated with ETs. LET ranked the highest for cardiac arrhythmia, myocardial infarction, and cardiac failure, while TAM exhibited a significantly high risk of stroke. ANA was the only therapy linked to a significant increase in reporting metabolic dysfunctions.

The observation of arthralgia as the most frequent reaction among women with breast cancer aligns with findings from our recent meta‐analysis of patients from developing countries and a review that mapped adverse effects of ETs [25, 26]. In addition to joint discomfort which can be disabling and limit daily activities, other common but more medically significant complications included depression, pulmonary embolism, uterine polyps, and dyspnea. Treatment‐induced depression is an emerging challenge in breast cancer survivorship because of its high prevalence and detrimental effects on cancer prognosis [27]. The risk of lung embolism and gynecological disorders (polyps) caused by TAM should also be carefully considered, especially given the evident treatment implication in thromboembolic events and uterine cancer development [28, 29]. Close vigilance of symptom distress and provision of supportive care strategies are essential to ensure patient safety and improve their quality of life.

Cardiovascular toxicities of breast cancer treatments have gained significant interest due to the observed increase in cardiac‐specific morbidities and mortalities among cancer survivors [13, 30]. Although some research has revealed previously unrecognized adverse cardiovascular effects of ETs, conflicting and mixed results are often reported [11, 12, 31, 32, 33]. For instance, participants in an international randomized clinical trial who were assigned to LET were more likely to develop myocardial ischemia than those who received TAM, and both groups showed a similar risk for cardiac failure outcomes [31]. In contrast, a large UK‐based cohort demonstrated an 86% increase in the incidence of heart failure and no differences in the risk of myocardial infarction or stroke in women receiving any AI compared to TAM users [12]. Similar findings were presented in another study of Italian women [32].

With regard to metabolic disorders, findings from published studies were inconsistent. A recent study suggested a positive correlation between the administration of AIs (unspecified by type) and the occurrence of hypertension, dyslipidemia, and diabetes [33]. However, none of these associations were detected in a meta‐analysis of clinical trials [11]. Additionally, Thomas et al. documented adverse metabolic effects linked to ETs, particularly noting negative impacts on lipid profiles and glucose tolerance [34]. Another comprehensive review evaluated the conflicting evidence surrounding metabolic outcomes of ET regimens [35].

A thorough update of the safety information in therapy package inserts is warranted to inform patients and alert healthcare providers to take appropriate precautions against the critical and potentially fatal (though unlabeled) problems detected in our study. These warnings should specifically involve routine monitoring of heart rate and myocardial injury during LET therapy, and the implementation of follow‐up measures for ANA users to detect changes in blood pressure, lipids, and glucose levels. This proactive approach will help to prevent, mitigate, and manage reactions before permanent damage occurs.

The selection of ET or decision on a regimen switch for better cancer care should be supported by knowledge of the patient's cardiac health status and an ongoing individualized benefit–risk assessment of treatment. Despite the identified adverse reactions, the life‐saving benefits of ETs must be reinforced. The administration of oral ET regimens in both early and advanced stages of breast cancer has significantly enhanced disease control, prevented cancer progression, and improved patient survival [9, 36].

Our study comprehensively examined the reactions of individual ET regimens and provided valuable insights into their adverse cardiovascular and metabolic effects in a real‐world setting using standard definitions of outcomes to ensure accuracy and avoid bias. However, this study has several limitations. Although the FAERS database has been a primary and useful platform for pharmacovigilance activities and has enabled the detection of several medication safety signals, underreporting of ADRs remains a major challenge [37, 38]. Therefore, the true risk of adverse events might be much higher than estimated. Conversely, overestimation is also possible, particularly when more severe events are disproportionately reported. Since submission to the pharmacovigilance database is voluntary, the reporting process is biased, and the data are exclusive to submitted cases not the entire population of ET users. It is also difficult to verify the accuracy of information, including the determined causal drug responsible for a specific event. Additionally, the same case may be reported multiple times resulting in duplicate entries. The number of reports is influenced by prescribing practices, usage patterns, as well as the time a medication has been on the market. Reporting rates may also vary based on the regulations in place at the time of approval. Finally, some critical variables, such as lifestyle behaviors, preexisting comorbidities, and co‐prescribed medications, could impact patient responses and introduce confounding effects on outcomes.

In conclusion, the global and cardiovascular safety of ETs was effectively evaluated using the FAERS database. Numerous broad‐spectrum adverse events were commonly reported among female breast cancer patients with the real‐world use of ETs. Our analysis identified signals of major cardiac reactions as well as significant increases in certain cardiovascular and metabolic abnormalities associated with specific regimens. These findings highlight the importance of close monitoring of cardiovascular function during therapy and ongoing assessment of the treatment benefit–risk ratio, particularly in women who are already at high risk of developing toxicities. The integration of cardiac surveillance strategies into routine oncology practice will help to achieve comprehensive patient care that optimizes cancer outcomes while minimizing cardiovascular injury.

## Author Contributions

**Shaimaa Elshafie:** conceptualization; methodology; data curation; formal analysis; writing – original draft. **Lorenzo Villa‐Zapata:** conceptualization; methodology; data curation; formal analysis; writing – review and editing. **Randall L Tackett:** writing – review and editing. **Iman Y Zaghloul:** writing – review and editing. **Henry N Young:** writing – review and editing.

## Conflicts of Interest

The authors declare no conflicts of interest.

## Supporting information


**Data S1.** Supplementary Information.

## Data Availability

Data that support the findings of this study were derived from the FDA public domain resource.
