# Supplementary material for: Cardiovascular and Metabolic Adverse Events of Endocrine Therapies in Women with Breast Cancer: A Disproportionality Analysis of Reports in the FDA Adverse Event Reporting System
Source: Cancer Med. 2024 Dec 30;14(1):e70548. doi: 10.1002/cam4.70548 (PMC11683672; doi:10.1002/cam4.70548)
Supplement: Supplementary file 1 — Data S1. Supplementary Information. [file CAM4-14-e70548-s001.docx]

**Supplementary Appendix**

**Definition of SMQs for studied outcomes**

| **Myocardial infarction** | |
| --- | --- |
| 'Acute cardiac event' | 'Blood creatine phosphokinase abnormal' |
| 'Acute coronary syndrome' | 'Blood creatine phosphokinase increased' |
| 'Acute myocardial infarction' | 'Cardiac ventricular scarring' |
| 'Angina unstable' | 'Coronary artery restenosis' |
| 'Blood creatine phosphokinase MB abnormal' | 'ECG electrically inactive area' |
| 'Blood creatine phosphokinase MB increased' | 'ECG signs of myocardial infarction' |
| 'Coronary artery embolism' | 'Electrocardiogram Q wave abnormal' |
| 'Coronary artery occlusion' | 'Electrocardiogram ST segment abnormal' |
| 'Coronary artery reocclusion' | 'Electrocardiogram ST segment elevation' |
| 'Coronary artery thrombosis' | 'Electrocardiogram ST-T segment elevation' |
| 'Coronary bypass thrombosis' | 'Electrocardiogram U wave inversion' |
| 'Coronary vascular graft occlusion' | 'Infarction' |
| 'Heart-type fatty acid-binding protein increased' | 'Myocardial necrosis marker increased' |
| 'Kounis syndrome' | 'Scan myocardial perfusion abnormal' |
| 'Myocardial infarction' | 'Vascular graft occlusion' |
| 'Myocardial necrosis' | 'Vascular stent occlusion' |
| 'Myocardial reperfusion injury' | 'Vascular stent thrombosis' |
| 'Myocardial stunning' |  |
| 'Papillary muscle infarction' |  |
| 'Periprocedural myocardial infarction' |  |
| 'Post procedural myocardial infarction' |  |
| 'Postinfarction angina' |  |
| 'Silent myocardial infarction' |  |
| 'Troponin I increased' |  |
| 'Troponin increased' |  |
| 'Troponin T increased' |  |

| **Cardiac failure** | | |
| --- | --- | --- |
| 'Acute left ventricular failure' | 'Artificial heart implant' | 'Left ventricular dysfunction' |
| 'Acute pulmonary oedema' | 'Atrial natriuretic peptide abnormal' | 'Left ventricular enlargement' |
| 'Acute right ventricular failure' | 'Atrial natriuretic peptide increased' | 'Lower respiratory tract congestion' |
| 'Cardiac asthma' | 'Bendopnoea' | 'Myocardial depression' |
| 'Cardiac failure' | 'Brain natriuretic peptide abnormal' | 'Myocardial strain imaging abnormal' |
| 'Cardiac failure acute' | 'Brain natriuretic peptide increased' | 'N-terminal prohormone brain natriuretic peptide abnormal' |
| 'Cardiac failure chronic' | 'Cardiac cirrhosis' | 'N-terminal prohormone brain natriuretic peptide increased' |
| 'Cardiac failure congestive' | 'Cardiac contractility decreased' | 'Neonatal dyspnoea' |
| 'Cardiac failure high output' | 'Cardiac contractility modulation therapy' | 'Nocturnal dyspnoea' |
| 'Cardiogenic shock' | 'Cardiac device implantation' | 'Oedema' |
| 'Cardiohepatic syndrome' | 'Cardiac device reprogramming' | 'Oedema blister' |
| 'Cardiopulmonary failure' | 'Cardiac dysfunction' | 'Oedema due to cardiac disease' |
| 'Cardiorenal syndrome' | 'Cardiac index decreased' | 'Oedema neonatal' |
| 'Chronic left ventricular failure' | 'Cardiac output decreased' | 'Oedema peripheral' |
| 'Chronic right ventricular failure' | 'Cardiac resynchronisation therapy' | 'Orthopnoea' |
| 'Congestive hepatopathy' | 'Cardiac ventriculogram abnormal' | 'Peripheral oedema neonatal' |
| 'Cor pulmonale' | 'Cardiac ventriculogram left abnormal' | 'Peripheral swelling' |
| 'Cor pulmonale acute' | 'Cardiac ventriculogram right abnormal' | 'Post cardiac arrest syndrome' |
| 'Cor pulmonale chronic' | 'Cardio-respiratory distress' | 'Prohormone brain natriuretic peptide abnormal' |
| 'Ejection fraction decreased' | 'Cardiomegaly' | 'Prohormone brain natriuretic peptide increased' |
| 'Heart failure with midrange ejection fraction' | 'Cardiothoracic ratio increased' | 'Pulmonary congestion' |
| 'Heart failure with preserved ejection fraction' | 'Central venous pressure increased' | 'Right ventricular diastolic collapse' |
| 'Heart failure with reduced ejection fraction' | 'Cerebrocardiac syndrome' | 'Right ventricular dilatation' |
| 'Hepatojugular reflux' | 'Chronic myocarditis' | 'Right ventricular dysfunction' |

| **Cardiac failure (continued)** | | |
| --- | --- | --- |
| 'Left ventricular failure' | 'Coronary sinus dilatation' | 'Right ventricular enlargement' |
| 'Low cardiac output syndrome' | 'Diastolic dysfunction' | 'Scan myocardial perfusion abnormal' |
| 'Neonatal cardiac failure' | 'Dilatation ventricular' | 'Stroke volume decreased' |
| 'Obstructive shock' | 'Dynamic cardiomyoplasty' | 'Surgical ventricular restoration' |
| 'Pulmonary oedema' | 'Dyspnoea paroxysmal nocturnal' | 'Systolic dysfunction' |
| 'Pulmonary oedema neonatal' | 'Global longitudinal strain abnormal' | 'Temporary mechanical circulatory support' |
| 'Radiation associated cardiac failure' | 'Heart and lung transplant' | 'Venous pressure increased' |
| 'Right ventricular ejection fraction decreased' | 'Heart transplant' | 'Venous pressure jugular abnormal' |
| 'Right ventricular failure' | 'Heart transplant failure' | 'Venous pressure jugular increased' |
| 'Ventricular failure' | 'Heart-lung transplant failure' | 'Ventricular assist device insertion' |
|  | 'Hepatic vein dilatation' | 'Ventricular compliance decreased' |
|  | 'Implantable cardiac monitor replacement' | 'Ventricular dysfunction' |
|  | 'Intracardiac pressure increased' | 'Ventricular dyssynchrony' |
|  | 'Jugular vein distension' | 'Ventricular outflow tract dredging' |
|  | 'Left ventricular diastolic collapse' | 'Wall motion score index abnormal' |
|  | 'Left ventricular dilatation' |  |

| **Arrhythmia** | | |
| --- | --- | --- |
| 'Accelerated idioventricular rhythm' | 'Bradyarrhythmia' | 'Electrocardiogram ambulatory abnormal' |
| 'Accessory cardiac pathway' | 'Bradycardia' | 'Electrocardiogram change' |
| 'Adams-Stokes syndrome' | 'BRASH syndrome' | 'Electrocardiogram delta waves abnormal' |
| 'Agonal rhythm' | 'Brugada syndrome' | 'Electrocardiogram P wave abnormal' |
| 'Anomalous atrioventricular excitation' | 'Bundle branch block bilateral' | 'Electrocardiogram PR prolongation' |
| 'Arrhythmia supraventricular' | 'Bundle branch block left' | 'Electrocardiogram PR shortened' |
| 'Arrhythmia' | 'Bundle branch block right' | 'Electrocardiogram QRS complex prolonged' |
| 'Arrhythmic storm' | 'Bundle branch block' | 'Electrocardiogram QT prolonged' |
| 'Atrial conduction time prolongation' | 'Cardiac arrest' | 'Electrocardiogram repolarisation abnormality' |
| 'Atrial escape rhythm' | 'Cardiac death' | 'Electrocardiogram RR interval abnormal' |
| 'Atrial fibrillation' | 'Cardiac fibrillation' | 'Electrocardiogram RR interval prolonged' |
| 'Atrial flutter' | 'Cardiac flutter' | 'Electrocardiogram RR interval shortened' |
| 'Atrial parasystole' | 'Cardiac telemetry abnormal' | 'Electrocardiogram U wave inversion' |
| 'Atrial standstill' | 'Cardio-respiratory arrest' | 'Electrocardiogram U wave present' |
| 'Atrial tachycardia' | 'Central bradycardia' | 'Electrocardiogram U-wave abnormality' |
| 'Atrioventricular block complete' | 'Cerebrocardiac syndrome' | 'Extrasystoles' |
| 'Atrioventricular block first degree' | 'Chronotropic incompetence' | 'Familial atrial fibrillation' |
| 'Atrioventricular block second degree' | 'Conduction disorder' | 'Fascicular block' |
| 'Atrioventricular block' | 'Congenital supraventricular tachycardia' | 'Frederick's syndrome' |
| 'Atrioventricular conduction time shortened' | 'Defect conduction intraventricular' | 'Heart alternation' |
| 'Atrioventricular dissociation' | 'Early repolarisation syndrome' | 'Heart rate abnormal' |
| 'Atrioventricular node dysfunction' | 'ECG P wave inverted' | 'Heart rate decreased' |
| 'Bezold-Jarisch reflex' | 'Ectopic atrial rhythm' | 'Heart rate increased' |
| 'Bifascicular block' | 'Electrocardiogram abnormal' | 'Heart rate irregular' |

| **Arrhythmia (continued)** | | |
| --- | --- | --- |
| 'Holiday heart syndrome' | 'Respiratory sinus arrhythmia magnitude abnormal' | 'Tachyarrhythmia' |
| 'Ictal bradycardia syndrome' | 'Respiratory sinus arrhythmia magnitude decreased' | 'Tachycardia paroxysmal' |
| 'Inappropriate sinus tachycardia' | 'Respiratory sinus arrhythmia magnitude increased' | 'Tachycardia' |
| 'Junctional ectopic tachycardia' | 'Retrograde p-waves' | 'Torsade de pointes' |
| 'Lenegre's disease' | 'Rhythm idioventricular' | 'Trifascicular block' |
| 'Long QT syndrome' | 'Sinoatrial block' | 'Ventricular arrhythmia' |
| 'Loss of consciousness' | 'Sinus arrest' | 'Ventricular asystole' |
| 'Nodal arrhythmia' | 'Sinus arrhythmia' | 'Ventricular dyssynchrony' |
| 'Nodal rhythm' | 'Sinus bradycardia' | 'Ventricular extrasystoles' |
| 'Pacemaker generated arrhythmia' | 'Sinus node dysfunction' | 'Ventricular fibrillation' |
| 'Pacemaker syndrome' | 'Sinus tachycardia' | 'Ventricular flutter' |
| 'Palpitations' | 'Sudden cardiac death' | 'Ventricular parasystole' |
| 'Parasystole' | 'Sudden death' | 'Ventricular pre-excitation' |
| 'Paroxysmal arrhythmia' | 'Supraventricular bradycardia' | 'Ventricular tachyarrhythmia' |
| 'Paroxysmal atrioventricular block' | 'Supraventricular extrasystoles' | 'Ventricular tachycardia' |
| 'Pulseless electrical activity' | 'Supraventricular tachyarrhythmia' | 'Wandering pacemaker' |
| 'Rebound tachycardia' | 'Supraventricular tachycardia' | 'Withdrawal arrhythmia' |
| 'Reperfusion arrhythmia' | 'Syncope' | 'Wolff-Parkinson-White syndrome' |

| **Stroke** | | |
| --- | --- | --- |
| 'Agnosia' | 'Capsular warning syndrome' | 'Cerebellar ischaemia' |
| 'Amaurosis fugax' | 'CARASIL syndrome' | 'Cerebellar microhaemorrhage' |
| 'Angiogram cerebral abnormal' | 'Carotid aneurysm rupture' | 'Cerebellar stroke' |
| 'Aphasia' | 'Carotid angioplasty' | 'Cerebral aneurysm perforation' |
| 'Balint's syndrome' | 'Carotid arterial embolus' | 'Cerebral aneurysm ruptured syphilitic' |
| 'Basal ganglia haematoma' | 'Carotid arteriosclerosis' | 'Cerebral angioplasty' |
| 'Basal ganglia haemorrhage' | 'Carotid artery aneurysm' | 'Cerebral arteriosclerosis' |
| 'Basal ganglia infarction' | 'Carotid artery bypass' | 'Cerebral arteriovenous malformation haemorrhagic' |
| 'Basal ganglia stroke' | 'Carotid artery disease' | 'Cerebral artery embolism' |
| 'Basilar artery aneurysm' | 'Carotid artery dissection' | 'Cerebral artery occlusion' |
| 'Basilar artery occlusion' | 'Carotid artery insufficiency' | 'Cerebral artery perforation' |
| 'Basilar artery perforation' | 'Carotid artery occlusion' | 'Cerebral artery restenosis' |
| 'Basilar artery stenosis' | 'Carotid artery perforation' | 'Cerebral artery stenosis' |
| 'Basilar artery thrombosis' | 'Carotid artery restenosis' | 'Cerebral artery stent insertion' |
| 'Benedikt's syndrome' | 'Carotid artery stenosis' | 'Cerebral artery thrombosis' |
| 'Brachiocephalic arteriosclerosis' | 'Carotid artery stent insertion' | 'Cerebral bypass surgery' |
| 'Brachiocephalic artery occlusion' | 'Carotid artery stent removal' | 'Cerebral cavernous malformation' |
| 'Brachiocephalic artery stenosis' | 'Carotid artery thrombosis' | 'Cerebral cyst haemorrhage' |
| 'Brain hypoxia' | 'Carotid blowout syndrome' | 'Cerebral endovascular aneurysm repair' |
| 'Brain injury' | 'Carotid endarterectomy' | 'Cerebral gas embolism' |
| 'Brain stem embolism' | 'Carotid revascularisation' | 'Cerebral haematoma' |
| 'Brain stem haematoma' | 'Central nervous system haemorrhage' | 'Cerebral haemorrhage foetal' |
| 'Brain stem haemorrhage' | 'Central pain syndrome' | 'Cerebral haemorrhage neonatal' |
| 'Brain stem infarction' | 'Cerebellar artery occlusion' | 'Cerebral haemorrhage' |
| 'Brain stem ischaemia' | 'Cerebellar artery thrombosis' | 'Cerebral haemosiderin deposition' |
| 'Brain stem microhaemorrhage' | 'Cerebellar atherosclerosis' | 'Cerebral infarction foetal' |
| 'Brain stem stroke' | 'Cerebellar embolism' | 'Cerebral infarction' |
| 'Brain stem thrombosis' | 'Cerebellar haematoma' | 'Cerebral ischaemia' |
| 'Brain stent insertion' | 'Cerebellar haemorrhage' | 'Cerebral microembolism' |
| 'CADASIL' | 'Cerebellar infarction' | 'Cerebral microhaemorrhage' |

| **Stroke (continued)** | | |
| --- | --- | --- |
| 'Cerebral microinfarction' | 'Embolic cerebral infarction' | 'Inner ear infarction' |
| 'Cerebral reperfusion injury' | 'Embolic stroke' | 'Internal capsule infarction' |
| 'Cerebral revascularisation' | 'Epidural haemorrhage' | 'Internal carotid artery deformity' |
| 'Cerebral septic infarct' | 'Extra-axial haemorrhage' | 'Intra-cerebral aneurysm operation' |
| 'Cerebral small vessel ischaemic disease' | 'Extradural haematoma evacuation' | 'Intracerebral haematoma evacuation' |
| 'Cerebral thrombosis' | 'Extradural haematoma' | 'Intracranial aneurysm' |
| 'Cerebral vascular occlusion' | 'Extraischaemic cerebral haematoma' | 'Intracranial artery dissection' |
| 'Cerebral vasoconstriction' | 'Foetal cerebrovascular disorder' | 'Intracranial haematoma' |
| 'Cerebral venous thrombosis' | 'Foville syndrome' | 'Intracranial haemorrhage neonatal' |
| 'Cerebral ventricular rupture' | 'Haemorrhage intracranial' | 'Intracranial tumour haemorrhage' |
| 'Cerebrovascular accident prophylaxis' | 'Haemorrhagic cerebellar infarction' | 'Intraventricular haemorrhage neonatal' |
| 'Cerebrovascular accident' | 'Haemorrhagic cerebral infarction' | 'Intraventricular haemorrhage' |
| 'Cerebrovascular disorder' | 'Haemorrhagic stroke' | 'Ischaemic cerebral infarction' |
| 'Cerebrovascular insufficiency' | 'Haemorrhagic transformation stroke' | 'Ischaemic stroke' |
| 'Cerebrovascular pseudoaneurysm' | 'Heidelberg classification' | 'Jugular vein embolism' |
| 'Cerebrovascular stenosis' | 'Hemianaesthesia' | 'Lacunar infarction' |
| 'Charcot-Bouchard microaneurysms' | 'Hemiasomatognosia' | 'Lacunar stroke' |
| 'Claude's syndrome' | 'Hemiataxia' | 'Lateral medullary syndrome' |
| 'Congenital hemiparesis' | 'Hemidysaesthesia' | 'Lateropulsion' |
| 'Cortical hand stroke' | 'Hemihyperaesthesia' | 'Malignant middle cerebral artery syndrome' |
| 'CSF bilirubin positive' | 'Hemihypoaesthesia' | 'Meningorrhagia' |
| 'CSF red blood cell count positive' | 'Hemiparaesthesia' | 'Metabolic stroke' |
| 'Delayed ischaemic neurological deficit' | 'Hemiparesis' | 'Middle cerebral artery stroke' |
| 'Diplegia' | 'Hemiplegia' | 'Migrainous infarction' |
| 'Dysarthria' | 'Hunt and Hess scale' | 'Millard-Gubler syndrome' |
| 'Embolic cerebellar infarction' | 'Hypoxic-ischaemic encephalopathy' | 'Modified Rankin score decreased' |

| **Stroke (continued)** | | |
| --- | --- | --- |
| 'Modified Rankin score increased' | 'Putamen haemorrhage' | 'Subdural haematoma' |
| 'Monoparesis' | 'Quadriparesis' | 'Subdural haemorrhage neonatal' |
| 'Monoplegia' | 'Quadriplegia' | 'Subdural haemorrhage' |
| 'Moyamoya disease' | 'Retinal artery occlusion' | 'Superficial siderosis of central nervous system' |
| 'NIH stroke scale abnormal' | 'Reversible cerebral vasoconstriction syndrome' | 'Temporal artery stenosis' |
| 'NIH stroke scale score decreased' | 'Reversible ischaemic neurological deficit' | 'Thalamic infarction' |
| 'NIH stroke scale score increased' | 'Right hemisphere deficit syndrome' | 'Thalamic stroke' |
| 'Occipital lobe stroke' | 'Ruptured cerebral aneurysm' | 'Thalamus haemorrhage' |
| 'Paralysis' | 'Septic cerebral embolism' | 'Thrombotic cerebral infarction' |
| 'Paraparesis' | 'Spinal artery embolism' | 'Thrombotic stroke' |
| 'Paraplegia' | 'Spinal artery thrombosis' | 'Transient ischaemic attack' |
| 'Paresis' | 'Spinal cord haematoma' | 'Vascular encephalopathy' |
| 'Parietal lobe stroke' | 'Spinal cord haemorrhage' | 'Vascular stent occlusion' |
| 'Perinatal stroke' | 'Spinal cord infarction' | 'Vascular stent stenosis' |
| 'Periventricular haemorrhage neonatal' | 'Spinal cord ischaemia' | 'Vein of Galen aneurysmal malformation' |
| 'Pituitary apoplexy' | 'Spinal epidural haematoma' | 'Vertebral artery aneurysm' |
| 'Pituitary haemorrhage' | 'Spinal epidural haemorrhage' | 'Vertebral artery arteriosclerosis' |
| 'Post cardiac arrest syndrome' | 'Spinal stroke' | 'Vertebral artery occlusion' |
| 'Post procedural stroke' | 'Spinal subarachnoid haemorrhage' | 'Vertebral artery perforation' |
| 'Post stroke depression' | 'Spinal subdural haematoma' | 'Vertebral artery stenosis' |
| 'Posthaemorrhagic hydrocephalus' | 'Spinal subdural haemorrhage' | 'Vertebral artery thrombosis' |
| 'Precerebral arteriosclerosis' | 'Stroke in evolution' | 'Vertebrobasilar artery dissection' |
| 'Precerebral artery aneurysm' | 'Subarachnoid haematoma' | 'Vertebrobasilar infarction' |
| 'Precerebral artery dissection' | 'Subarachnoid haemorrhage neonatal' | 'Vertebrobasilar insufficiency' |
| 'Precerebral artery embolism' | 'Subarachnoid haemorrhage' | 'Vertebrobasilar stroke' |
| 'Precerebral artery occlusion' | 'Subclavian steal syndrome' | 'Visual agnosia' |
| 'Precerebral artery thrombosis' | 'Subcortical stroke' | 'Visual midline shift syndrome' |
| 'Pseudo-occlusion of internal carotid artery' | 'Subdural haematoma evacuation' | 'Weber's syndrome' |
| **Hypertension** | | |
| 'Accelerated hypertension' | 'Blood pressure orthostatic increased' | 'Hypertensive cerebrovascular disease' |
| 'Aldosterone urine abnormal' | 'Blood pressure systolic abnormal' | 'Hypertensive crisis' |
| 'Aldosterone urine increased' | 'Blood pressure systolic increased' | 'Hypertensive emergency' |
| 'Angiotensin converting enzyme abnormal' | 'Catecholamine crisis' | 'Hypertensive encephalopathy' |
| 'Angiotensin converting enzyme increased' | 'Catecholamines urine abnormal' | 'Hypertensive end-organ damage' |
| 'Angiotensin I abnormal' | 'Catecholamines urine increased' | 'Hypertensive heart disease' |
| 'Angiotensin I increased' | 'Dialysis induced hypertension' | 'Hypertensive nephropathy' |
| 'Angiotensin II abnormal' | 'Diastolic hypertension' | 'Hypertensive urgency' |
| 'Angiotensin II increased' | 'Diuretic therapy' | 'Labile blood pressure' |
| 'Angiotensin II receptor type 1 antibody positive' | 'Eclampsia' | 'Labile hypertension' |
| 'Blood aldosterone abnormal' | 'Ectopic aldosterone secretion' | 'Malignant hypertension' |
| 'Blood aldosterone increased' | 'Ectopic renin secretion' | 'Malignant hypertensive heart disease' |
| 'Blood catecholamines abnormal' | 'Endocrine hypertension' | 'Malignant renal hypertension' |
| 'Blood catecholamines increased' | 'Epinephrine abnormal' | 'Maternal hypertension affecting foetus' |
| 'Blood pressure abnormal' | 'Epinephrine increased' | 'Mean arterial pressure increased' |
| 'Blood pressure ambulatory abnormal' | 'Essential hypertension' | 'Metabolic syndrome' |
| 'Blood pressure ambulatory increased' | 'Gestational hypertension' | 'Metanephrine urine abnormal' |
| 'Blood pressure diastolic abnormal' | 'HELLP syndrome' | 'Metanephrine urine increased' |
| 'Blood pressure diastolic increased' | 'Hyperaldosteronism' | 'Neurogenic hypertension' |
| 'Blood pressure fluctuation' | 'Hypertension neonatal' | 'Nocturnal hypertension' |
| 'Blood pressure inadequately controlled' | 'Hypertension' | 'Norepinephrine abnormal' |
| 'Blood pressure increased' | 'Hypertensive angiopathy' | 'Norepinephrine increased' |
| 'Blood pressure management' | 'Hypertensive cardiomegaly' | 'Normetanephrine urine increased' |
| 'Blood pressure orthostatic abnormal' | 'Hypertensive cardiomyopathy' | 'Orthostatic hypertension' |

| **Hypertension (continued)** | | |
| --- | --- | --- |
| 'Page kidney' | 'Renal sympathetic nerve ablation' | 'Superimposed pre-eclampsia' |
| 'Postoperative hypertension' | 'Renal vascular resistance increased' | 'Supine hypertension' |
| 'Pre-eclampsia' | 'Renin abnormal' | 'Syndrome Z' |
| 'Prehypertension' | 'Renin increased' | 'Systolic hypertension' |
| 'Primary hyperaldosteronism' | 'Renin-angiotensin system inhibition' | 'Tyramine reaction' |
| 'Procedural hypertension' | 'Renovascular hypertension' | 'White coat hypertension' |
| 'Pseudoaldosteronism' | 'Retinopathy hypertensive' | 'Withdrawal hypertension' |
| 'Renal artery revascularisation' | 'Secondary aldosteronism' |  |
| 'Renal hypertension' | 'Secondary hypertension' |  |

| **Dyslipidemia** | |
| --- | --- |
| 'Acquired mixed hyperlipidaemia' | 'Lipid metabolism disorder' |
| 'Apolipoprotein B/Apolipoprotein A-1 ratio increased' | 'Lipids abnormal' |
| 'Atherogenic index of plasma abnormal' | 'Lipids decreased' |
| 'Atherogenic index of plasma decreased' | 'Lipids increased' |
| 'Atherogenic index of plasma increased' | 'Lipoprotein (a) abnormal' |
| 'Autoimmune hyperlipidaemia' | 'Lipoprotein (a) decreased' |
| 'Blood cholesterol abnormal' | 'Lipoprotein (a) increased' |
| 'Blood cholesterol decreased' | 'Lipoprotein abnormal' |
| 'Blood cholesterol esterase increased' | 'Lipoprotein increased' |
| 'Blood cholesterol increased' | 'Lipoprotein metabolism disorder' |
| 'Blood triglycerides abnormal' | 'Low density lipoprotein abnormal' |
| 'Blood triglycerides decreased' | 'Low density lipoprotein decreased' |
| 'Blood triglycerides increased' | 'Low density lipoprotein increased' |
| 'Diabetic dyslipidaemia' | 'Metabolic syndrome' |
| 'Dyslipidaemia' | 'Non-high-density lipoprotein cholesterol decreased' |
| 'Familial high density lipoprotein deficiency' | 'Non-high-density lipoprotein cholesterol increased' |
| 'Familial hypertriglyceridaemia' | 'Primary hypercholesterolaemia' |
| 'Fat overload syndrome' | 'Remnant hyperlipidaemia' |
| 'High density lipoprotein abnormal' | 'Remnant-like lipoprotein particles increased' |
| 'High density lipoprotein decreased' | 'Total cholesterol/HDL ratio abnormal' |
| 'High density lipoprotein increased' | 'Total cholesterol/HDL ratio decreased' |
| 'Hypercholesterolaemia' | 'Total cholesterol/HDL ratio increased' |
| 'Hyperlipidaemia' | 'Type I hyperlipidaemia' |
| 'Hypertriglyceridaemia' | 'Type II hyperlipidaemia' |
| 'Hypertriglyceridaemic waist phenotype' | 'Type IIa hyperlipidaemia' |
| 'Hypo HDL cholesterolaemia' | 'Type IIb hyperlipidaemia' |
| 'Hypotriglyceridaemia' | 'Type III hyperlipidaemia' |
| 'Intermediate density lipoprotein decreased' | 'Type IV hyperlipidaemia' |
| 'Intermediate density lipoprotein increased' | 'Type V hyperlipidaemia' |
| 'LDL/HDL ratio decreased' | 'Very low density lipoprotein abnormal' |
| 'LDL/HDL ratio increased' | 'Very low density lipoprotein decreased' |
| 'Lecithin-cholesterol acyltransferase deficiency' | 'Very low density lipoprotein increased' |

| **Hyperglycemia** | | |
| --- | --- | --- |
| 'Abnormal loss of weight' | 'Blood triglycerides increased' | 'Diabetic wound' |
| 'Abnormal weight gain' | 'Body mass index decreased' | 'Diabulimia' |
| 'Acidosis' | 'Body mass index increased' | 'Euglycaemic diabetic ketoacidosis' |
| 'Acquired generalised lipodystrophy' | 'Carbon dioxide combining power abnormal' | 'Fructosamine increased' |
| 'Adiponectin decreased' | 'Carbon dioxide combining power decreased' | 'Fulminant type 1 diabetes mellitus' |
| 'Alpha hydroxybutyric acid increased' | 'Central obesity' | 'Gestational diabetes' |
| 'Altered state of consciousness' | 'Coma' | 'Glucose tolerance decreased' |
| 'Anti-GAD antibody positive' | 'Continuous glucose monitoring' | 'Glucose tolerance impaired in pregnancy' |
| 'Anti-IA2 antibody positive' | 'Dehydration' | 'Glucose tolerance impaired' |
| 'Anti-insulin antibody increased' | 'Depressed level of consciousness' | 'Glucose tolerance test abnormal' |
| 'Anti-insulin antibody positive' | 'Diabetes complicating pregnancy' | 'Glucose urine present' |
| 'Anti-insulin receptor antibody increased' | 'Diabetes mellitus inadequate control' | 'Glycated albumin increased' |
| 'Anti-insulin receptor antibody positive' | 'Diabetes mellitus' | 'Glycated serum protein increased' |
| 'Anti-islet cell antibody positive' | 'Diabetes with hyperosmolarity' | 'Glycosuria during pregnancy' |
| 'Anti-zinc transporter 8 antibody positive' | 'Diabetic arteritis' | 'Glycosuria' |
| 'Blood 1,5-anhydroglucitol decreased' | 'Diabetic coma' | 'Glycosylated haemoglobin abnormal' |
| 'Blood cholesterol increased' | 'Diabetic coronary microangiopathy' | 'Glycosylated haemoglobin increased' |
| 'Blood glucose abnormal' | 'Diabetic hepatopathy' | 'Hepatogenous diabetes' |
| 'Blood glucose fluctuation' | 'Diabetic hyperglycaemic coma' | 'Hunger' |
| 'Blood glucose increased' | 'Diabetic hyperosmolar coma' | 'Hypercholesterolaemia' |
| 'Blood insulin abnormal' | 'Diabetic ketoacidosis' | 'Hyperglycaemia' |
| 'Blood insulin decreased' | 'Diabetic ketoacidotic hyperglycaemic coma' | 'Hyperglycaemic crisis' |
| 'Blood lactic acid increased' | 'Diabetic ketosis' | 'Hyperglycaemic hyperosmolar nonketotic syndrome' |
| 'Blood osmolarity increased' | 'Diabetic metabolic decompensation' | 'Hyperglycaemic seizure' |

| **Hyperglycemia (continued)** | | |
| --- | --- | --- |
| 'Hyperglycaemic unconsciousness' | 'Ketoacidosis' | 'Postprandial hypoglycaemia' |
| 'Hyperlactacidaemia' | 'Ketonuria' | 'Pseudodiabetes' |
| 'Hyperlipidaemia' | 'Ketosis-prone diabetes mellitus' | 'Slow response to stimuli' |
| 'Hyperosmolar state' | 'Ketosis' | 'Steroid diabetes' |
| 'Hyperphagia' | 'Lactic acidosis' | 'Syndrome Z' |
| 'Hypertriglyceridaemia' | 'Latent autoimmune diabetes in adults' | 'Thirst' |
| 'Hypoglycaemia' | 'Lipids increased' | 'Type 1 diabetes mellitus' |
| 'Hypoinsulinaemia' | 'Loss of consciousness' | 'Type 2 diabetes mellitus' |
| 'Hypoinsulinism' | 'Maternally inherited diabetes and deafness' | 'Type 3 diabetes mellitus' |
| 'Impaired fasting glucose' | 'Metabolic acidosis' | 'Underweight' |
| 'Impaired insulin secretion' | 'Metabolic syndrome' | 'Unresponsive to stimuli' |
| 'Increased appetite' | 'Monogenic diabetes' | 'Urine glucose/creatinine ratio abnormal' |
| 'Increased insulin requirement' | 'Neonatal diabetes mellitus' | 'Urine glucose/creatinine ratio decreased' |
| 'Indeterminate glucose tolerance' | 'Neonatal hyperglycaemia' | 'Urine glucose/creatinine ratio increased' |
| 'Insulin autoimmune syndrome' | 'New onset diabetes after transplantation' | 'Urine ketone body present' |
| 'Insulin resistance' | 'Obesity' | 'Weight decreased' |
| 'Insulin resistant diabetes' | 'Overweight' | 'Weight increased' |
| 'Insulin therapy' | 'Pancreatogenous diabetes' | 'Wolfram syndrome' |
| 'Insulin tolerance test abnormal' | 'Polydipsia' |  |
| 'Insulin-requiring type 2 diabetes mellitus' | 'Polyuria' |  |
